# Editorial: Multi-omics insights into exercise-induced molecular adaptations

**DOI:** 10.3389/fspor.2026.1992834

**Published:** 2026-10-06

**Authors:** Lixian He, Haibo Wang, Qingzheng Kang, Liang Zhang

**Affiliations:** 1 Hubei Superior Discipline Group of Exercise and Brain Science from Hubei Provincial, Wuhan Sports University, Wuhan, China; 2 Institute of Biomedical and Health Science, School of Biomedical and Health, Anhui Science and Technology University, Chuzhou, Anhui, China; 3 The Eighth Affiliated Hospital, Sun Yat-sen University, Shenzhen, China

**Keywords:** exercise, multi-omics, genomics, proteomics, PTMs, transcriptomics

## Introduction

Exercise is renowned for its wide-ranging health benefits, contributing to the reduction of cardiometabolic diseases, neurodegenerative disorders, and cancer risks while enhancing metabolic and immune health. The molecular response to exercise is not a linear cascade but a multi-dimensional, temporally orchestrated interplay across biological layers. Genomics provides the blueprint, transcriptomics the transcriptional response, proteomics/PTM-omics the functional execution, and metabolomics the systemic readout. Among these, PTM-omics stands out as the most dynamic and least explored frontier, bridging rapid environmental sensing with long-term molecular memory. This Research Topic, “Multi-omics insights into exercise-induced molecular adaptations,” assembles studies that collectively map this complexity—spanning static genetic blueprints, transcriptional dynamics, proteomic regulation, and metabolic outcomes—while emphasizing the transition from descriptive profiling toward mechanistic understanding and translational application.

1. Genomics – decoding inherited athletic potential

Human physical performance and training adaptation result from complex interactions, including genetic and environmental factors (1). Common genomic sequence variation contributes substantially to inter-individual differences in muscular fitness, cardiorespiratory capacity, metabolic responsiveness to acute exercise, and long-term adaptation to training. Key performance-related phenotypes show similarly strong genetic contributions: baseline aerobic capacity (60%), anaerobic power (70%–90%), and muscular strength (50%–70%) are all substantially heritable (2). Su et al. conduct a systematic review and meta-analysis on the PPARGC1A Gly482Ser polymorphism, revealing a clear population-specific association: the Gly/Gly genotype confers a significant advantage for elite endurance and power athlete status in Caucasians, but this effect is absent or even reversed in Asian cohorts. This work highlights that genomic markers are not universal; their predictive value depends on ethnic background, necessitating ancestry-aware frameworks in sports genetics and talent identification. These genomic insights set the stage for understanding how transcriptional programs translate genetic predisposition into functional responses.

2. Transcriptomics – capturing dynamic gene expression reprogramming

Moving from DNA to RNA, exercise induces rapid and tissue-specific transcriptional reprogramming that underpins metabolic and structural adaptation (3). Xie et al. leverage publicly available transcriptomic datasets to identify differentially expressed genes in diabetic cardiomyopathy models, intersecting them with oxidative stress-related gene sets. Through bioinformatic network analysis, they pinpoint protein C (PROC) as a hub gene whose expression is rescued by aerobic exercise. This transcriptomics-guided approach provides a powerful strategy to nominate novel exercise-responsive targets for subsequent mechanistic validation. The advent of single-cell and single-nucleus RNA sequencing is now revolutionizing this landscape (4). Recent studies have leveraged these technologies to dissect exercise-induced transcriptional dynamics at unprecedented resolution—from immune cell reprogramming in endurance runners to cell-type-specific regulatory circuits in skeletal muscle. Yet transcriptional changes alone do not reveal protein-level regulation, necessitating a deeper look into the proteome.

3. Proteomics and PTM-omics – the functional bridge between genes and phenotypes

While transcriptomics reveals which genes are turned on or off, it cannot predict protein abundance, activity, or interaction—functions that operate at the protein level. The Molecular Transducers of Physical Activity Consortium (MoTrPAC) represents the most comprehensive effort to bridge this gap through systematic multi-omic profiling of exercise responses (5). “MoTrPAC's publicly accessible data provide an essential resource for dissecting the molecular mechanisms of exercise adaptation at the protein level, directly supporting the premise that proteomics serves as the functional bridge between static genetic blueprints and dynamic physiological outcomes. Complementing these proteomic insights, post-translational modifications (PTMs) add an additional layer of regulatory complexity. Shen et al. systematically review the emerging concept of PTM-mediated regulatory encoding, demonstrating that PTMs act as dynamic information carriers that integrate spatial and temporal signals across organelles, tissues, and organ systems. Among the diverse PTM repertoire, several classes stand out for their direct relevance to exercise adaptation:

Phosphorylation acts as the first responder to stress. Exercise activates multiple kinases, triggering phosphorylation cascades through AMPK and mTOR—pathways that exhibit distinct temporal dynamics, providing a molecular rationale for periodized training design (6, 7).

Ubiquitination acts as the gatekeeper of protein quality control. Ubiquitination marks proteins for proteasomal degradation or directs them through autophagy and DNA repair pathways (8). Exercise regulates this system bidirectionally: acute stress upregulates E3 ligases to clear damaged proteins, while chronic training suppresses proteolysis and promotes net muscle accretion—ensuring efficient remodeling without excessive breakdown (9).

Glycosylation serves as a metabolic sensor and functional coupler, directly reflecting nutrient availability. Its bidirectional regulation under exercise exemplifies the “adaptive homeostasis” principle, where transient elevation may protect against excessive phosphorylation, while long-term normalization reflects metabolic recovery (10, 11).

Lactylation, pyruvylation, and beyond represent emerging frontiers. Lactylation links glycolysis-derived lactate to epigenetic regulation, with HIIT inducing persistent histone modifications that outlast circulating lactate clearance—a modality-specific PTM signature that offers a molecular basis for precision exercise prescription. Pyruvylation, succinylation, malonylation and vitcylation remain largely unexplored in exercise contexts, yet hold immense potential to reveal how metabolic intermediates directly sculpt the proteomic and epigenetic landscape during and after exercise.

These emerging PTMs represent uncharted territories with immense potential to reveal how metabolic intermediates directly sculpt the proteomic and epigenetic landscape during and after exercise (12–14).

4. Metabolomics and systemic integration – the physiological outcome

At the apex of the molecular hierarchy, metabolomics captures the cumulative output of genomic, transcriptional, and proteomic/PTM regulation. Xu et al. propose a “bidirectional threshold theory,” framing exercise as a double-edged sword. They trace how primary mechanical and organelle stress signals propagate through AMPK/mTOR/NAD + energy-sensing networks, ultimately manifesting as systemic metabolic shifts—glucose utilization, fatty acid oxidation, and ketone body production (6). This systemic integration is further coordinated by endocrine, myokine, and immune mediators, determining whether the organism adapts or maladapts.

## Conclusion

The Research Topic “Multi-omics insights into exercise-induced molecular adaptations” presents important contributions to our understanding of how physical activity reshapes the biological landscape across multiple molecular layers. Putting together all the findings presented in this Research Topic, it is possible to verify that exercise adaptation emerges from a dynamic and temporally orchestrated interplay among genomics, transcriptomics, proteomics/PTM-omics, and metabolomics. From population-specific genetic markers influencing athlete status, through transcriptomics-guided identification of novel exercise-responsive genes, to proteomic and PTM-omic discoveries revealing how phosphorylation, acetylation, glycosylation, lactylation, and other modifications dynamically regulate protein function—and finally to metabolomic readouts that capture systemic physiological outcomes—these multi-layered insights collectively demonstrate that exercise writes its adaptive legacy through a coordinated molecular language. These multi-omics findings may offer insights relevant to the development of more precise, evidence-based exercise strategies for individuals and practitioners, with potential implications for physical performance, metabolic health, and quality of life.
